# Delayed effect of Kinesio Taping on neuromuscular performance, balance, and lower limb function in healthy individuals: a randomized controlled trial

**DOI:** 10.1590/bjpt-rbf.2014.0161

**Published:** 2016-03-22

**Authors:** Caio A. A. Lins, Daniel T. Borges, Liane B. Macedo, Karinna S. A. Costa, Jamilson S. Brasileiro

**Affiliations:** 1 Laboratório de Análise da Performance Neuromuscular (LAPERN), Departamento de Fisioterapia, Universidade Federal do Rio Grande do Norte (UFRN), Natal, RN, Brazil

**Keywords:** torque, electromyography, bandages

## Abstract

**Background:**

Kinesio Taping (KT) is an elastic bandage that aims to improve neuromuscular performance, although there is no consensus as to its benefits.

**Objective:**

To analyze the immediate and delayed effects of KT on the neuromuscular performance of the femoral quadriceps, on balance, and lower limb function in healthy subjects.

**Method:**

This is a randomized controlled trial. Thirty-six women with a mean age of 22.2±3.6 years and BMI of 22.5±2.3 Kg/m^2^ were divided into three groups: control, with ten minutes of rest (control, n=12), application of Kinesio Taping without tension (placebo, n=12) and with tension (KT, n=12) on the quadriceps. The primary outcome was isokinetic performance, while secondary outcomes were the single-hop test, one-footed static balance, and electromyographic activity. The evaluations were carried out in five stages: 1) before application of KT, 2) immediately after the application of KT, 3) after 24h, 4) after 48h, and 5) after 72h. Mixed ANOVA was used to determine differences between groups.

**Results:**

There was no change in one-footed static balance, electromyographic activity of the VL in the lower limb function, nor in isokinetic performance between groups.

**Conclusion:**

KT promotes neither immediate nor delayed changes in neuromuscular performance of the femoral quadriceps in healthy women.

## Bullet points

- The study evaluated immediate and delayed effects of the application of Kinesio Taping.
- KT did not change immediate or delayed neuromuscular performance.
- KT application effects do not depend on the time/duration of application.
- The results do not support the hypothesis that the application of KT results in performance improvement.

## Introduction

Kinesio Taping (KT) is an elastic bandage developed by Kenzo Kase. According to its creator, it has specific features, ranging from its design to its elongation^1^, that improve functional performance. In practice, this technique has been widely used by healthy people in order to prevent injuries and increase neuromuscular performance, seeking better performance during physical activities, whether at the professional or amateur level^2^.

KT consists of a thin elastic tape which can be stretched up to 50% of its original length, resulting in lower restriction compared to conventional tapes^1^, thereby proposing to increase joint stability and improve muscular performance^3^. However, the mechanisms by which the application of KT reaches such goals are not well understood. One such mechanism would be by increasing muscle activity during the implementation of KT through neurofacilitation, where the tactile stimulation provided by the tape activates cutaneous receptors, thus promoting alpha motor neuron stimulation^4^. Furthermore, due to its characteristics, the bandage could provide increased interstitial space, promoting better blood and lymph flow in the region^1^ ^,^ ^5^ ^-^ ^7^.

In this context, the effect of applying KT has been the subject of research to evaluate its influence on both balance and function of the lower limbs, as well as on muscle activation (EMG) and strength (dynamometry) in patients and in healthy people, but with conflicting results^8^ ^-^ ^15^. Recently, a meta-analysis on the effect of KT on increasing muscle strength showed that its implementation does not promote improvement in healthy adults^16^. Another meta-analysis on the influence of KT on the treatment and prevention of sports injuries showed that this technique has little beneficial effect on muscle strength, muscle activation, or active range of motion^17^. However, the studies included in both meta-analyses are classified as being of moderate methodological quality and only a few of them found significant effects. In addition, the authors make it clear that more research needs to be conducted, particularly blind randomized controlled studies that include a placebo group.

Two other systematic reviews investigated the clinical effects of KT and reported that there are few high-quality studies and therefore insufficient evidence to support the use of this technique in clinical practice^18^ ^,^ ^19^. A study by Słupik et al.^20^ noted that there was no increase in the electromyographic activity of the vastus medialis (VM) during isometric contraction of the knee extensors immediately after applying KT to this muscle. Nevertheless, the same study noted an increase in electromyographic activity of the VM at 24 and 72 hours after applying KT and 24 hours after removal of the bandage. These findings raise a hypothesis of the possible delayed effects of applying KT, suggesting that an adjustment period would be needed in the application technique in order to meet the expected goals of healthy people. However, the same study did not use a placebo or control group, in addition to only observing the effect of KT on one variable.

Thus, there is no consensus in the literature about the real effects of KT, although this technique is being widely used by healthy people seeking better performance during physical activities. In addition, few studies have evaluated its chronic effects on neuromuscular performance, both on patients and on healthy people. Given the above, this study aimed to analyze the immediate and delayed effects of KT application on isokinetic knee extensor performance, electromyographic activity of the vastus lateralis (VL), one-footed static balance, and lower limb function for healthy subjects.

## Method

### Subjects

This is a randomized controlled trial consisting of 36 healthy women with a mean age of 22.2±3.6 years and body mass index (BMI) of 22.5±2.3 Kg/m^2^. They were non-probabilistically recruited and randomly distributed using the website www.randomization.com. Only female subjects were included due to the large biomechanical differences that occur between genders. The inclusion criteria were: age between 18 and 28 years; being recreationally active^21^; hip, knee, and ankle joint integrity; no history of musculoskeletal injury in the last 6 months; no previous surgical history of their lower limbs; uncorrected neurological, vestibular, visual, and/or auditory deficits; allergy to the adhesive material. Subjects who incorrectly executed the assessment procedures or missed any evaluations were excluded from the study.

The participants received information about the research objectives and signed a free and informed consent form, according to Resolution 466/12 of the National Health Council and the Declaration of Helsinki. The study was approved by the Ethics Committee of Universidade Federal do Rio Grande do Norte (UFRN), Natal, RN, Brazil (protocol number 752.302). This study was registered at www.clinicaltrials.gov under registration number NCT02431910.

### Procedures

A pilot study was conducted in order to adjust all the research procedures and to train the researchers involved. Two evaluators participated in the study: evaluator 1 was responsible for carrying out the evaluation of all of the subjects, while the second evaluator was responsible for implementing the intervention. However, due to the presence of a group that did not apply the bandage, the subjects and evaluator 1 were not blinded to the intervention performed.

Initially, all of the subjects filled out an evaluation form with anthropometric data (age, weight, height, and BMI), personal information, and questions about physical activity frequency. Next, they performed a warm up on a stationary bicycle for five minutes (ErgoFit Cycle 167, Ergo-Fit, Pirmasens, Germany), with a 15W load at a constant speed of 20 km/h, and with their seat adjusted to the height of the greater trochanter of the femur.

After the warm up, the isokinetic performance evaluation was performed, considered as the primary outcome of the study. In addition, one-footed static balance, lower limb function, and VL electromyographic activity were also assessed and considered as secondary outcomes. The evaluations were always conducted using the non-dominant limb, which was set from the subject's account by asking which leg they use to kick a ball.

The evaluations were performed at five distinct time points: before the intervention protocol (pre), immediately after (post), and 24h, 48h, and 72h after the intervention protocol. The last evaluation (72h) was performed 24h after the removal of KT.

### Isokinetic performance evaluation

To carry out this evaluation, the subject was placed in the sitting position in the chair of a computerized isokinetic dynamometer (Biodex Multi-Joint System 4™, Biodex Medical Systems Inc., Shirley, NY, USA). The dominant thigh was fixed by a strap, as were the pelvis and thorax region. On the non-dominant limb, the dynamometer rotation axis was aligned with the lateral epicondyle of the femur and the lever arm was adjusted to the distal region of the leg and fixed at 5 cm above the medial malleolus of the ankle. The gravity correction factor was carried out by the dynamometer itself, adjusted by the weight of the relaxed leg at 30° of knee flexion.

The isokinetic performance evaluation was performed by five concentric knee extension contractions at 60°/s. This evaluation started from 90° flexion up to full extension of the knee and recorded the peak torque normalized by body weight (PT/BW), expressed as percentage and average power. The return to flexed position was done passively.

During the evaluation, verbal encouragement and visual feedback were provided by the computer. To become familiarized with the equipment, the subjects performed three submaximal contractions at 60º/s, followed by a 60-second interval until the start of testing.

### One-footed static balance evaluation

For this evaluation, subjects were assessed on a computerized baropodometry platform (Eclipse 3000, Guy-Capron SAS, Montchanin, France) with a 40×40 cm surface and acquisition frequency of 20Hz. They were positioned standing on the platform to support the non-dominant limb and with their knee flexed at 20° (considering 0° to be full knee extension), verified by a universal goniometer. The subject was then instructed to keep their head in a neutral position looking at a fixed point, with their spine erect and upper limbs supported on their hips. The dominant lower limb remained with the hip at 0° and the knee at 90° flexion. Data acquisition time was ten seconds. The assessment was repeated three times, with the average of the two repetitions that showed the least fluctuation being considered for analysis. The rest time was one minute between each test, and the analyzed variables were the displacement velocity of the pressure center in the anteroposterior and mediolateral directions.

### Lower limb function evaluation

The single-hop test was performed, considered testing measures of functional performance^22^. They were instructed to start the hop without the support of the contralateral limb to avoid impulse movements.

The subjects were encouraged to perform a single hop as far as possible without any type of footwear, and the hallux-hallux distance was measured using a tape measure. To allow for a comparison of values between the subjects, the data were normalized as a function of the height of each subject (hop distance/height × 100).

The test was repeated twice, and the further of the two measurements was recorded. For the hop to be considered valid, the subject should remain balanced for two seconds after completing the hop and the contralateral limb could not touch the ground. One minute of rest was allowed between tests.

### Electromyographic activity records

For electromyographic activity analysis of the VL muscle, the skin was shaved and cleansed with 70% alcohol before electrode placement. An 8-channel signal conditioning module with 16-bit resolution (TeleMyo Transmitter, Noraxon Inc., Scottsdale, AZ, USA) was used for signal acquisition and common-mode rejection ratio (CMRR) >100 Db. Signals were captured on a sampling frequency set at 1500 Hz, filtered at a frequency between 10 and 500 Hz and amplified 1000 times. Signals were captured using passive adhesive surface electrodes (Noraxon Inc.) 4 cm long and 2.2 cm wide, separated by an inter-electrode distance of 2 cm. The electrode was placed on the VL muscle belly, according to recommendations of the SENIAM (Surface Electromyography for the Non-Invasive Assessment of Muscles) project^23^. The software myoResearch 3.2 (Noraxon Inc.) was used for analysis of the digital signals.

The electromyographic activity recording was conducted simultaneously with the knee extensor torque evaluation. Therefore, the average RMS analysis during the concentric evaluation was considered as the electromyographic signal of higher torque from the five recorded on the isokinetic dynamometer, being carried out with a 1-s window during contraction for the analysis. Normalization was performed by the RMS peak value during maximal voluntary isometric contraction, as the subjects were instructed to perform two knee extension contractions at an angle of 60° flex for 5 seconds, with a 60-second rest interval between them. The contraction that generated the most torque was used for normalization.

### Interventions

After the baseline assessment, the subjects were randomly assigned to one of three groups. The second evaluator applied the protocol according to randomization: control group (n=12) - remained 10 minutes at rest (time required for applying the bandages in the other groups); placebo group (n=12) - application of KT (kinesio tex Gold®) to the femoral quadriceps (FQ) muscle without tension; and KT group (n=12) - application of KT on the FQ muscle with tension.

Subjects from the KT group were submitted to KT application on the FQ of the non-dominant limb as suggested by Kase et al.^1^ to increase muscle performance. Thus, the bandage was applied to the rectus femoris (RF), VL, and VM longitudinally, from proximal to distal. For the RF muscle, the proximal anchor was applied 5 cm below the anterior superior iliac spine and the distal anchor was placed on the upper edge of the patella. For the VL muscle, the proximal and distal anchors were placed on the greater trochanter of the femur and on the lateral edge of the patella, respectively. As for the VM muscle, the proximal anchor was placed on the middle third of the medial thigh region and the distal anchor on the medial edge of the patella. For the three muscles in question, the anchors were applied with 0% tension and the therapeutic area (area between the anchors) was followed on the belly of muscles with 50% tension, in order to promote greater muscle activation^1^. This application was carried out with the subjects standing on one foot, with the hip of the non-dominant limb at 0° and the knee flexed, as suggested by Lins et al.^14^, keeping the muscle in a stretched position. For the placebo group, the same protocol was followed, except that the application of the bandage was maintained at 0% tension on the anchor and also in the treatment zone.

### Statistical analysis

Based on initial values obtained from a pilot study conducted with 15 subjects, a sample of 36 subjects with 12 in each group was adequate to detect a clinically significant difference of 12.0% between groups, assuming a standard deviation of 41.0 for the PT/BW outcome during the concentric contraction. A statistical power of 80%, an alpha of 5%, and a loss rate of 10% were considered for the sample calculation. The sample size calculation was performed for the ANOVA repeated measures statistical test with interactions between groups. The software Gpower3.1 was used for the calculation.

Statistical analysis was performed using the Statistical Package for the Social Sciences software (SPSS) version 20.0. The normal distribution of data and homogeneity of variance were verified by the Kolmogorov-Smirnov (KS) and Levene tests, respectively. Estimates of average effect (differences between groups) for all variables were calculated using the ANOVA mixed model. This analysis model incorporated the intervention groups (control, placebo, and kinesio taping), time (pre, post, 24h, 48h, and 72h), and the group × time interaction. When a significant F value was found, the Bonferroni post-hoc test was applied in order to identify the differences. A significance level of 5% was adopted for all statistical analyses (P<0.05), which were conducted by an independent researcher.

## Results

**Figure 1:**
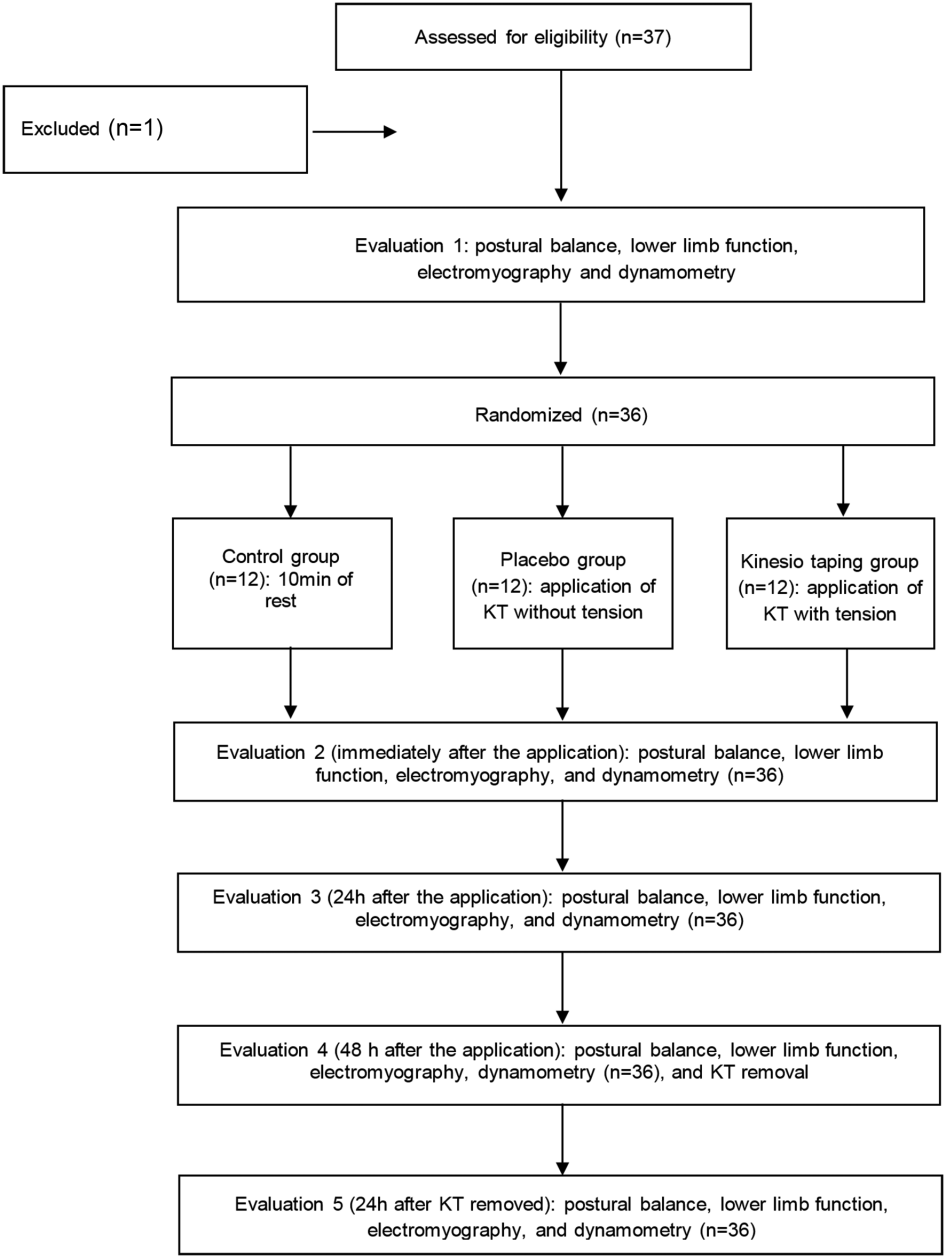
Study flow diagram.

One subject was excluded from the study because she felt pain at the time of initial evaluation (Figure 1).

Table 1 shows the homogeneity for the analyzed variables between the groups at baseline.

**Table 1:** Mean values and standard deviation (SD) of age, height, body mass index (BMI), anteroposterior velocity (A/P VEL), mediolateral velocity (M/L VEL), single hop, RMS of VL muscle (RMS VL), peak torque normalized by body weight (PT/BW), and average power of all groups evaluated at baseline.

**VARIABLES**	**CONTROL**	**PLACEBO**	**KINESIO TAPING**
	**n=12**	**n=12**	**n=12**
**Age** (years)	21.4 (3.6)	22.3 (3.8)	23.3 (3.1)
**Height** (m)	1.63 (0.06)	1.64 (0.03)	1.65 (0.07)
**BMI** (Kg/m^2^)	22.3 (2.4)	22.7 (2.3)	22.5 (2.3)
**A/P VEL** (mm/s)	11.1 (1.6)	10.2 (1.6)	11.5 (3.0)
**M/L VEL** (mm/s)	5.1 (1.2)	4.6 (0.7)	5.2 (1.0)
**Single Hop** (%)	72.7 (7.6)	72.8 (11.7)	76.2 (9.6)
**RMS VL** (%)	62.2 (14.1)	63.6 (23.5)	58.2 (16.8)
**PT/BW** (%)	254.7 (33.9)	231.3 (32.3)	240.2 (47.4)
**Power** (W)	82.3 (14.5)	76.4 (19.4)	84.1 (24.4)

Data expressed as mean and standard deviation (SD).

Table 2 shows the mean values and standard deviation of the analyzed variables at the five time points of evaluation (pre, post, 24h, 48h, and 72h) for the three groups.

**Table 2:** Mean values and standard deviation (SD) of the variables: anteroposterior velocity (a/p vel), mediolateral velocity (m/l vel), single hop, RMS of VL muscle (RMS VL), peak torque normalized by body weight (PT/BW) and average power, in five stages of evaluation (pre, post, 24h, 48h, and 72h), of all groups.

	**CONTROL** **(n=12)**					**PLACEBO** **(n=12)**					**KINESIO TAPING** **(n=12)**				
**Variables**	**Mean (SD)**					**Mean (SD)**					**Mean (SD)**				
	**PRE**	**POST**	**24h**	**48h**	**72h**	**PRE**	**POST**	**24h**	**48h**	**72h**	**PRE**	**POST**	**24h**	**48h**	**72h**
a/p Vel (mm/s)	11.0 (1.7)	10.8 (1.7)	10.6 (2.1)	10.8 (1.8)	10.5 (2.8)	10.1 (1.6)	9.8 (1.7)	9.5 (2.4)	10.5 (2.0)	10.0 (2.4)	11.5 (3.0)	10.0 (2.8)	10.1 (2.6)	10.5 (2.8)	9.6 (1.8)
m/l Vel (mm/s)	5.1 (1.2)	5.1 (1.0)	4.8 (1.1)	5.3 (1.2)	5.0 (1.5)	4.6 (0.7)	4.2 (0.6)	4.4 (0.8)	4.9 (0.8)	4.6 (1.0)	5.2 (1.0)	4.6 (1.0)	4.8 (1.0)	5.1 (1.2)	4.5 (0.9)
Single hop (%)	72.7 (7.6)	79.4 (7.4)	81.2 (8.3)	81.7 (7.5)	82.0 (9.5)	72.8 (11.7)	77.3 (11.2)	81.0 (11.2)	84.0 (9.9)	84.6 (10.5)	78.3 (9.6)	82.2 (9.9)	83.0 (10.7)	86.3 (9.3)	84.5 (11.4)
RMS VL (%)	62.2 (14.1)	65.4 (14.1)	75.0 (13.0)	67.8 (7.2)	70.2 (8.6)	63.6 (23.5)	66.1 (18.1)	66.0 (18.6)	66.2 (23.1)	69.0 (21.4)	58.1 (16.8)	63.0 (20.3)	59.0 (17.6)	63.0 (18.4)	61.7 (18.7)
PT/BW (%)	254.7 (33.9)	250.2 (30.7)	253.5 (35.7)	247.4 (46.6)	256.0 (37.1)	231.3 (32.3)	230.0 (31.0)	224.0 (41.0)	245.6 (42.1)	242.4 (23.2)	240.2 (47.4)	239.1 (47.4)	230.3 (42.7)	245.7 (44.3)	246.6 (42.7)
Power (W)	82.3 (14.5)	84.6 (13.0)	85.5 (17.3)	85.7 (19.6)	88.1 (19.0)	76.3 (19.4)	74.1 (12.7)	76.4 (14.7)	81.2 (14.7)	82.8 (11.0)	84.0 (24.4)	81.8 (21.8)	85.6 (23.8)	86.6 (22.2)	86.7 (21.4)

Data expressed as mean and standard deviation (SD).

Table 3 presents the analysis between groups for the comparisons post and 24h after the intervention, while Table 4 also shows the analysis between groups, as well as the comparisons for 48h and 72h after the intervention. No difference was detected between the groups in the assessments for all variables: PT/BW (F=1.015, p=0:42); average power (F=0.534, p=0.76); anterior-posterior displacement velocity (F=1.050, p=0.40) and medial-lateral displacement velocity (F=0.697, p=0.69); distance of single hop (F=1.442, p=0.18); and VL muscle RMS (F=1.226, p=0.28).

**Table 3:** Differences between groups immediately and 24 hours after intervention in all groups (control, placebo, and Kinesio Taping) for all analyzed variables: anteroposterior velocity (a/p vel), mediolateral velocity (m/l vel), single hop, RMS of VL muscle (RMS VL), peak torque normalized by body weight (PT/BW), and average power.

**Variables**		**Mean differences between groups** **Confidence interval (95% CI)**												
**Immediately after intervention** **(95% CI), p**								**24 hours after intervention** **(95% CI), p**						
	**Control** **vs** **Placebo**		**p**	**Control** **vs** **Kinesio**	**p**	**Kinesio** **vs** **Placebo**	**p**	**Control** **vs** **Placebo**	**p**	**Control** **vs** **Kinesio**	**p**	**Kinesio** **vs** **Placebo**	**p**
a/p Vel (mm/s)	1.1 (–1.3-3.5)		0.78	0.8 (–1.4-3.0)	0.90	0.2 (–2.0-2.6)	0.95	1.1 (–1.5-3.7)	0.89	0.4 (–1.9-2.8)	0.96	0.6 (–1.8-3.2)	0.94
m/l Vel (mm/s)	0.9 (–0.1-1.9)		0.08	0.5 (–0.3-1.4)	0.40	0.3 (–0.6-1.3)	0.94	0.3 (–0.7-1.4)	0.92	0.1 (–0.9-0.9)	0.98	0.3 (–0.6-1.4)	0.90
Single hop (%)	2.1 (–8.2-12.4)		0.99	–2.8 (–12.2-6.7)	0.96	4.9 (–5.1-14.8)	0.68	0.2 (–10.7-11.0)	0.99	-1.8 (–11.8-8.2)	0.90	1.9 (–8.6-12.5)	0.92
RMS VL (%)	–0.6 (–19.9-18.6)		0.96	2.4 (–15.3-20.2)	0.92	–3.1 (–21.7-15.6)	0.98	8.9 (–8.8-26.8)	0.64	16.0 (–0.3-32.4)	0.06	–7.0 (–24.2-10.2)	0.93
PT/BW (%)	21.3 (–20.5-63.2)		0.62	11.0 (–27.3-49.5)	0.99	10.2 (–30.2-50.7)	0.90	29.5 (–13.5-72.6)	0.28	23.2 (16.4-62.8)	0.44	6.3 (–35.3-48.0)	0.99
Power (W)	10.4 (–7.8-28.7)		0.48	2.7 (–14.0-19.5)	0.95	7.7 (–10.0-25.4)	0.84	9.1 (–12.0-30.2)	0.85	–0.4 (–19.4-19.3)	0.95	9.1 (–11.2-29.5)	0.80

Mean differences and confidence intervals (95% CI).

**Table 4:** Differences between groups at 48 hours and 72 hours after intervention in all groups (control, placebo and Kinesio Taping) for all analyzed variables: anteroposterior velocity (a/p vel), mediolateral velocity (m/l vel), single hop, RMS of VL muscle (RMS VL), peak torque normalized by body weight (PT/BW), and average power.

**Variables**		**Mean differences between groups** **Confidence interval (95% CI)**												
**48 hours after intervention** **(95% CI), p**								**72 hours after intervention** **(95% CI), p**						
	**Control** **vs** **Placebo**		**p**	**Control** **vs** **Kinesio**	**p**	**Kinesio** **vs** **Placebo**	**p**	**Control** **vs** **Placebo**	**p**	**Control** **vs** **Kinesio**	**p**	**Kinesio** **vs** **Placebo**	**P**
a/p Vel (mm/s)	0.2 (–2.2-2.7)		0.92	0.2 (–2.0- 2.5)	0.95	0.02 (–2.4-2.4)	0.98	0.5 (–2.0-3.1)	0.91	0.9 (–1.3-3.3)	0.89	–0.4 (–2.9-2.0)	0.96
m/l Vel (mm/s)	0.4 (–0.8-1.6)		0.90	0.1 (–0.9-1.3)	0.91	0.2 (–0.9-1.4)	0.96	0.4 (–0.8-1.7)	0.98	0.4 (–0.7-1.6)	0.99	–0.02 (–1.3-1.2)	0.97
Single hop (%)	–2.3 (–12.0-7.3)		0.98	–4.6 (–13.4-4.2)	0.59	2.2 (–7.0-11.5)	0.90	–2.6 (–14.0-8.7)	0.92	–2.5 (–13.0-8.0)	0.97	–0.1 (–11.1-10.9)	0.93
RMS VL (%)	1.6 (–17.0-20.2)		0.84	4.8 (–12.2-22.0)	0.94	–3.2 (–21.2-14.7)	0.92	1.2 (–17.0-19.5)	0.95	8.5 (–8.3-25.3)	0.63	–7.2 (–25.0-10.4)	0.92
PT/BW (%)	1.8 (–46.2-49.9)		0.98	1.7 (–42.4-45.8)	0.90	0.1 (–46.3-46.5)	0.96	13.5 (–25.8-52.8)	0.92	9.3 (–26.8-45.4)	0.99	4.1 (–33.8-42.2)	0.98
Power (W)	4.5 (–16.6-25.6)		0.91	–0.8 (–20.2-18.6)	0.95	5.3 (–15.1-25.7)	0.99	5.3 (–14.4-25.0)	0.96	1.4 (–16.7-19.5)	0.92	3.9 (–15.2-23)	0.92

Mean differences and confidence intervals (95% CI).

## Discussion

This study aimed to evaluate the immediate and delayed effects of KT application on the neuromuscular knee extension performance in one-footed static balance and lower limb function of healthy subjects. The results indicated that the application of KT does not promote immediate or delayed changes to displacement velocity of the pressure center in the anteroposterior or mediolateral directions, the distance of the single hop, the electromyographic amplitude of VL, the normalized peak torque or the average power of knee extensors.

Corroborating the results of this study, Nunes et al.^24^ evaluated the effects of applying KT to the sural triceps on vertical jump, drop jump, and single-leg stance in athletes and did not observe changes in these variables. Lins et al.^14^ found no change in distance for the single and triple hop in healthy subjects after applying KT to the FQ. In addition, Huang et al.^25^ analyzed vertical jump height 30 minutes after applying KT to the sural triceps in healthy subjects and found no significant change in that variable. However, unlike the present study, Nakajima and Baldridge^26^ observed that the application of KT to the ankle did not change the vertical jump height, but increased the dynamic postural control in healthy subjects. They say it is possible that the tension supplied by KT may have increased the neural feedback during ankle motion, improving balance, but the tactile stimulus was not strong enough to increase muscle power while performing the jump.

Thus, we suggest that the application of KT in healthy people does not influence one-footed static balance or lower limb function. A possible explanation for these results could be the application of KT only to the quadriceps muscle, since other muscles and joints, such as the hip and ankle, are also involved in these activities. Therefore, the application to just one muscle group cannot provide enough incentive to change these variables in healthy women. It is worth emphasizing that, unlike other studies, this study evaluated the delayed effect of KT on these variables and found no significant changes even after 48 hours of application and 24 hours after its removal, thus demonstrating that “an adaptation period” is not necessary for the application technique to achieve the expected goals, as suggested in previous studies^20^ ^,^ ^27^.

A study by Słupik et al.^20^ noted that there was no increase in the electromyographic activity of the VM immediately after the application of KT to this muscle. However, they observed an increase in VM electromyographic activity 24h and 72h after KT application and 24h after removal of the bandage. Mohammadi et al.^27^ observed an increase in grip strength immediately after KT application to the elbow flexors and extensors and 90 minutes after application of the technique. The results of these studies raise the hypothesis of possible delayed effects of KT application on neuromuscular performance, which differs from the results of this study where we observed no significant changes in any of the variables in any of the assessed time points.

Studies evaluating the delayed effects of the technique are rare and have different methodologies, especially relating to the duration of KT application. Generally, the immediate effect of KT on neuromuscular performance is evaluated, as noted by Lins et al.^14^ and Oliveira et al.^15^. Those studies noted the immediate effect of KT application on the FQ in healthy subjects and in subjects undergoing reconstruction of the anterior cruciate ligament, respectively, verifying that applying the technique did not significantly change the electromyographic activity of the VL or the isokinetic knee extensor performance.

In this study, the application of KT did not promote any changes in the analyzed parameters, suggesting that the tactile stimulation promoted by KT did not sufficiently alter neuromuscular performance in healthy people. In addition, our study evaluated the delayed effects of KT on these variables, showing that there were also no significant changes compared to previous values, therefore there is no need for an adjustment period for the application technique to promote greater activation of the proposed mechanisms of action, i.e. neurofacilitation^4^ and increase in local blood flow^1^. Thus, we suggest that there is no evidence to support the application of this technique for this population or in order to improve athletic performance.

It is worth emphasizing that the results of this study should be limited to healthy and active women who practice recreational physical activity. Thus, it is suggested that further studies are conducted to evaluate the chronic effects of KT on function, balance, and neuromuscular performance of patients in the rehabilitation process.

## Conclusion

The results of this study suggest that the application of KT to the quadriceps muscle is not able to promote immediate or delayed changes to neuromuscular performance, balance, or lower limb function in healthy, active women.
